# Dual Consciousness and Allied States

**Published:** 1899-02-25

**Authors:** James Allan Philip


### Hospital Clinics and Medical Progress.

### DUAL CONSCIOUSNESS AND ALLIED STATES.

By James Allan Philip, M.A., M.D.

Cerebral instability shows itself in protean forms —madness, imbecility, idiocy, eccentricity, hysteria, epilepsy, and a number of minor conditions which we call neurotic.

There are naturally, therefore, relations between these conditions; they have characteristics in common, and some of them may manifest certain symptoms which are similar or identical.

I propose to cite a few examples of more or less pronounced dual consciousness arising from very different causes, which seem to me to tend to show that it is a very common condition, although often very transient. The most interesting phenomenon in this condition seems to me the continuity of memory from one "attack" to the next, which seems also to be present in the hypnotic condition, and sometimes also in somnambulism, in acute mania, &c. In many cases of acute mania there is an apparent resemblance to the condition in dreaming, when fantastic visions unchecked by reason pass in succession through the brain. We can, I think, form a chain of relations between the least abnormal cerebral conditions, and the most grave, from sleep, the somnambulism of adolescence, the hysteria of puberty, to acute mania, or the delirium of fever, &c.

Dr. Proust, of Paris, described, in 1870, a case of dual consciousness. The patient was a barrister, of a neurotic family, who was extremely susceptible to hypnotising agencies, so much so that the fixed gaze of the judge, when the patient was pleading a case, or his own reflection in a mirror, were sufficient to send him into a state of hypnotism. On more than one occasion he disappeared from home and was absent for periods extending to some weeks. Once he travelled into the country and visited an uncle, a bishop, in whose house he behaved very strangely, breaking his furniture, &c. He was also locked up for some offence. Of his life during these abnormal conditions he had no recollection afterwards; but that he was in a state allied to the hypnotic was shown by his remembering what had occurred when he had been purposely hypnotised. Here we seem to have crossed the borderland between the hypnotic and other conditions closely allied to insanity.

In the next case, probably due to epilepsy, there seems to be no relation to hypnotism, but dual consciousness seems to have appeared in a manner very similar to certain post-epileptic phenomena. Some epileptics have periods of what is styled epileptic mania, during which they become moody and suspicious, and delusions appear which sometimes lead to terrible tragedies, of which the patient appears to have no recollection when he has returned to his normal state of mind. Although in the case I am about to describe there is no history of any apparent mental disturbance of such a character, yet it seems to me that the attacks I shall refer to are of a similar character.

A young man came to see me one evening, and told me that he was in an extraordinary position, that he found himself, an hour before, seated on the quay, ignorant of where he was or of how he got there. He could tell me nothing about himself, his name, his home, or his occupation. He was tall, powerfully built, and fresh coloured. He had no appearance of having been drinking, or of having been taking any narcotics, but his pupils were widely dilated, and his pulse was quick and rather full. He was silent and passive in his manner, answering questions readily enough, but less disturbed by his situation than might have been expected. Cards, letters, &c., found in his pockets gave him no clue to his name, &c. I sent him to an hotel, and gave him a sleeping draught (no opiate). He appeared to sleep soundly that night, and next morning was much the same as the night before. He accompanied me in a passive manner to some hotels which I visited, hoping to learn where he had been living. A visiting card in his pocket seemed to him to recall something, and he recognised his name on an account. By midday he said he felt very exhausted, and returned to his hotel. In the afternoon his manner had quite changed; he was alert and brisk, and remembered everything about himself, but was quite unable to say how or when he had come here. I sent him home next day.* He told me that he suffered from frequent attacks of giddiness, particularly when on his way to business, and had been under treatment for epilepsy. A few months before he went to Dover, stayed there a fortnight, giving out that he was an engineer engaged at Folkestone, to which place he went daily. He found himself at Folkestone one day unable to account for his being there; went to Dover, as he had a return half of a ticket from it, and to the hotel whose name was on a bill in his pocket. He did something similar more recently, but came to his senses more quickly. He appeared to have no recollection of his life during these periods, only learning what had happened from others. It may be noted that when I saw him first he was in a third period, when he remembered neither his normal nor his abnormal life. I do not know whether this has been observed in cases of epileptic mania. My next case I mention merely to show the probable existence of continuity of memory during the abnormal periods, which seems very similar to more typical cases of dual consciousness.

A young man, somewhat moody, silent and neurotic, spoiled by a neurotic mother, had attacks of pseudo convulsions and spasmodic rigidity, which were evidently hysterical. These lasted sometimes half an hour, or an hour. During the earlier attacks he showed no irritability when checked in his struggling, but this grew, and in consequence of this he was sometimes secured to the bed. Later, as soon as any attempts to restrain him were made he flew into a furious passion, kicked, bit, and tore at those holding him, and ground his teeth and roared with rage. I do not think that he had any recollection of these attacks after they were over, but it seemed to me that during an attack he had a recollection of the rough handling he had received during previous ones, and was ready to resent a repetition of it. Appropriate treatment for hysteria rapidly cured him.

The last case to which I shall refer was described by

* The dilatation of the pupils had disappeared.

Dr. Albert Wilson before the Clinical Society in 1898. It was one of these mixed cases of hysteria and mania, which are so difficult to classify. A girl, arriving at puberty, had influenza, followed by meningitis, and then intolerance of light and sound. Acute mania followed, with hallucinations and delusions of persecution, muscular twitchings and opisthotomos. The mania subsided after a few weeks, and then the symptoms I wish to refer to occurred. When quietly amusing herself, or otherwise occupied, she would call out, "It is coming," and push away her work, toys, &c. Her legs began to shake, and then she turned a somersault and appeared in a new personality, the details of which are described in The Hospital for March 6th. She talked more like a young child, and was totally altered in manner. This condition lasted for from ten to fifty minutes, and then she appeared dazed, but began playing or occupying herself as if there had been no interruption. Here continuity of memory was observed, and the similarity of the symptoms to those of the hysterical young man would make it likely that continuity of memory existed in his case also. Consequently, the most striking feature of dual consciousness exists in many conditions not hitherto regarded as included in it.

It would not be difficult to multiply examples of more or less typical dual consciousness, but I venture to think that those I have cited will show that it is not so rare as one has considered it, and that it appears in many forms of mental disturbance. They also show the impossibility of classifying cerebral disorders when heredity is a leading factor, since, apart from the fact that parents suffering from one form of neurosis may transmit the nervous instability in quite a different form, these apparently widely different affections are more or less closely allied to each other.

